# Meta-analysis and systematic review of physical activity on neurodevelopment disorders, depression, and obesity among children and adolescents

**DOI:** 10.3389/fpsyg.2022.940977

**Published:** 2022-11-30

**Authors:** Sanying Peng, Yuan Fang, Ahmad Tajuddin Othman, Jinghong Liang

**Affiliations:** ^1^Department of Physical Education, Hohai University, Nanjing, China; ^2^School of Educational Studies, Universiti Sains Malaysia, Penang, Malaysia; ^3^College of International Languages and Cultures of Hohai University, Nanjing, China; ^4^Department of Maternal and Child Health, School of Public Health, Sun Yat-sen University, Guangzhou, China

**Keywords:** physical activity, physical and mental health, children and adolescents, meta-analysis, randomized controlled trial

## Abstract

**Background:**

No consensus on whether physical activity (PA) is related to physical and mental health among pediatric population remains has been reached to date. To further explore their association, our study assessed the effect of PA on physical and mental health of children and adolescents through a systematic review and meta-analysis of randomized controlled studies (RCTs).

**Methods:**

Several databases(Web of science, PubMed, Embase, Cochrane Central register of controlled trials, CINAHL) were searched from inception to 1st, December 2020 without language restrictions.

**Results:**

38,236 records were identified primitively and 31 included studies with 1,255 participants eventually met our inclusion criteria, all of which exhibited a relatively low-moderate risk of bias of overall quality. In regard to mental health, the administration of PA, compared with the control group, led to moderate improvements in Autism Spectrum Disorder(ASD)[Standard mean difference (SMD) = −0.50, Confidence interval(CI): −0.87, −0.14)] and depression(SMD = −0.68, CI: −0.98, −0.38) among children and adolescents. Similarly, significant result was observed in obesity (SMD = −0.58, CI: −0.80, −0.36). No significant differences were observed in Attention deficit hyperactivity disorder (ADHD) (SMD = −0.29, CI: −0.59, 0.01).

**Conclusion:**

Altogether, PA may have a beneficial effect on children and adolescents with ASD, depression and obesity; nevertheless, there is insufficient evidence to confirm its efficacy in ADHD. More large-scale population based randomized controlled trials are needed to explore more reliable evidence between them.

## Introduction

Adolescence is a period accompanied by various changes, in which both physical and mental diseases may negatively impact the development of youth. Such changes may further pose a threat to their health and even the stability of the whole society. Epidemiological evidence revealed that globally approximately 20% of children and adolescents suffer from mental health disorders, (WHO, 2013) which are often represented by anxiety, attention deficit hyperactivity disorder (ADHD), as well as depression, the most frequently diagnosed disorder (Satcher, 2000). These mental health issues are important determinants of health in a person's later life. However, currently, there exists no reliable evidence for the explanation of the etiologic factors of ASD and ADHD, as well as depression and anxiety, two universal health mental disorders afflicting children and adults alike (Wang et al., 2016; Naghavi et al., 2017). Moreover, it is predicted that the following decades will see a globally rapid growth of obesity among the youth, (Wang and Lobstein, 2006; Reilly and Kelly, 2011). The global prevalence of teens with excess weight and obesity increased by 6.9% from 1980 to 2013 (Ng, 2014), and reached 17.1% now (Laing and Rochester, 2005; Ogden and Tabak, 2005). Therefore, obesity is not only a growing problem troubling the youth, but also results in heavy healthcare burden and financial costs, which calls for concern from the policy makers (WHO, 2021).

The prevention and treatment of obesity and mental disorders among children and adolescents, whose prevalence is even more serious in the US, are two global public health challenges (Evans et al., 2005; Grunbaum et al., 2012). In this context, it is necessary to find preventive interventions and reliable therapy to reduce the symptom and eventually improve their mental wellbeing. The effect of Physical activity (PA) on mental health has been an important research topic in the area of public health and psychological interdisciplinary (Brown et al., 2013; Spruit et al., 2016).

Currently, reliable evidence on the association between PA and physical and mental health suggests that moderate-to-vigorous physical activity probably has a positive impact on physical and mental health (Biddle and Asare, 2019; Kelley et al., 2019). Several empirical meta-analyses focusing on the relationship between PA and mental health suggested that PA seemed to contribute to a significant but small improvement in mental diseases such as depression, anxiety, and social disorders in children (Lubans et al., 2016; Zhou et al., 2019). Although the effectiveness of PA has been investigated by many previous studies, they are not without limitations, which may influence the generalization of the findings. First of all, deficiencies in methodology affect the reliability of the results. Some previous reviews only looked at the effect of PA with a single indicator (Pearson et al., 2014) or included assorted controls (Placebo, intervention as usual or wait list) in one study (Azevedo et al., 2016). Some included and pooled different types of trials (RCT, quasi-experimental trials, and quasi-control trials) for their analyses (Howells et al., 2019). Second, some of the existing studies draw controversial conclusions regarding whether PA has significantly improved the overall health of children and adolescents (Kelley et al., 2003; Howells et al., 2019), possibly due to a lack of large-scale population-based trials. As a result, the association is not persuasive and related evidence is fragmented as multiple health outcomes have not been discussed together in one study. Considering the contradictory results in previous studies and the new evidence for this topic, we need to update our systematic review to evaluate and synthesize the newest evidence on the effects of PA on children's and adolescents's mental health. Therefore, as an updated and comprehensive meta-analysis in contrast to the previous literature-based studies, our study extends the aforementioned objectives and intends to examine the efficacy of the PA intervention on neurodevelopment disorders, depression and obesity of children and adolescents ranging from 6 to 18 years old.

## Methods

### Literature search

There is no restriction on language, and only randomized controlled trials (RCTs), with no limitation on whether they are parallel or over-cross design, which examine the association between PA and neurodevelopment disorders, depression, and obesity among children and adolescents were considered. A comprehensive and exhaustive search strategy was built by comprising Medical Subject Headings (MeSH) related to “Children”, “Adolescent”, “Teenager”, “Physical activity”, and “Randomized controlled trials”, and keywords including “Students”, “Teenagers”, “Youth”, “Enrollment”, “Acute exercise”, “Isometric exercise”, “Resistance exercise”, “Mind-body exercise”, “Exercise Training”, “Randomized controlled trial”, and “Pilot trial” were used, incorporating Boolean operators, to systematically search Web of Science, PubMed, Embase, Cochrane Central Register of Controlled Trials, and CINAHL from their inception to 1^st^ December 2020. The details of the search strategies based on each database are introduced in the Supplementary material. Additionally, recursive hand-search was performed by reviewing the studies from large-scale specialized conferences and retrieving the bibliographies of similar systematic reviews or meta-analyses to avoid omission. The studies whose topic seemed relevant were retained for further full-text review. Our analysis was performed in accordance with the Preferred Reporting Items for Systematic Reviews and Meta-Analyses (PRISMA)-2020 declaration (Page et al., 2021). All the records collected above were compiled and managed in EndNote X9 Software (Thompson ISI Research Soft, Philadelphia, Clarivate Analytics).

### Study inclusion criteria and study selection

The studies included should simultaneously meet the following criteria:

(1) Children or adolescents aged below 11 years old or teenagers aged from 12 to 18 years old with at least one of the selected health-related outcomes (neurodevelopment disorders, depression, and obesity) were participants of the original study;
(2) Any type of randomized controlled studies with either a parallel or over-cross design;
(3) The total sample size of each included study should be over 20 due to the fact that a relatively smaller sample size might yield an underpowered result;
(4) The outcomes include a battery of or any combination of diseases mentioned before;
(5) The children or adolescents had a diagnosis of any physical and mental disorder (neurodevelopment disorders, depression, and obesity) based on standardized diagnostic interviews;
(6) The full-text study was published in any index journal.
  (1) Additionally, studies would be excluded if they were reports, protocol studies, or full-text that were unpublished or presented without an abstract.

Two investigators independently assessed eligible studies by screening titles and abstracts along with, if necessary, the full text of potential articles. In addition, a manual search was performed to examine relevant research in case any potentially useful studies were left out. Disagreement during the screening process was solved by consensus, or judged by an experienced expert in the field.

### Data extraction

Based on the Cochrane Consumers and Communication Review Group's data extraction template (Shuster, 2011), essential publication information (the first author, publication year, and recruitment area), participants' characteristics (total sample size, proportion of gender, and follow-up time), and available data for analysis (outcome measures and quality of studies) were documented for each eligible study. Any discrepancies were settled by discussion.

### Quality of individual studies

The risk of bias (ROB) in all included studies was assessed and classified according to the Cochrane Risk of Bias tool (Higgins and Green, 2011), which consists of seven domains: random sequence generation, allocation concealment, blinding of participants and personnel, blinding of outcome assessment, incomplete outcome data, selective reporting, and other bias. The evaluation of ROB was performed in Review Manager (Version 5.3). We evaluated each of the seven domains independently and scored each study with a high, low, or unknown risk of bias. Furthermore, funnel plots and an Egger's test for each outcome were generated to determine whether there were publication biases (Egger, 1997). Two authors of this review independently assessed the quality of each included study according to the designated criteria.

### Statistical analyses

In view of the fact that we constricted our meta-analysis to a continuous outcome, we calculated the standard mean differences (SMDs) with a fixed effects approach (DerSimonian-Laird estimator) to pool the corresponding estimates, along with their 95% confidence intervals (CIs) as a measure of an uncertain estimate (Higgins and Green, 2011). Cochran's *Q-*test and the *P*-value were both used to judge whether there is heterogeneity among the included studies, as well as the *I-*squared method. An *I-*square value higher than 50% indicates more heterogeneity. We introduced the analyzing tool of a radar plot for forming a characteristics relation between each outcome. Subsequently, based on the primary outcome and various covariates which were rated as pre-established concomitant variables, a sequence of subgroup analyses was performed for exploring the sources of heterogeneity more accurately among these studies and the difference of each item based on the summary results. The sequence of areas considered in our model is as follows: Recruitment Region (Asia, Africa, and Australia vs. America and Europe); Publication years (Publication year ≥ 2012 vs. Publication years < 2012); Grade group (Primary vs. Middle and Secondary vs. High school); Total sample size (Sample size ≥ 40 vs. Sample size < 40); Age group [children group (aged from 6 to 12) vs. adolescent (aged from 13 to 18)]; ROB Quality (Low and Unclear ROB vs. High ROB); Duration weeks (Weeks ≥ 12 vs. Weeks < 12); Male to female ratio (Ratio ≥1 vs. Ratio < 1); Implementation site (School vs. Specific area); and Type of obese (Obesity vs. Overweight). Two investigators independently analyzed the available data. Any divergence during the process of overall analyses was resolved by consensus and referring back to the original studies. The above analyses were carried out by means of STATA, SE-64, Version 15.0 (Stata Corp, College Station, Tex).

## Results

### Literature selection

**Figure 1:**
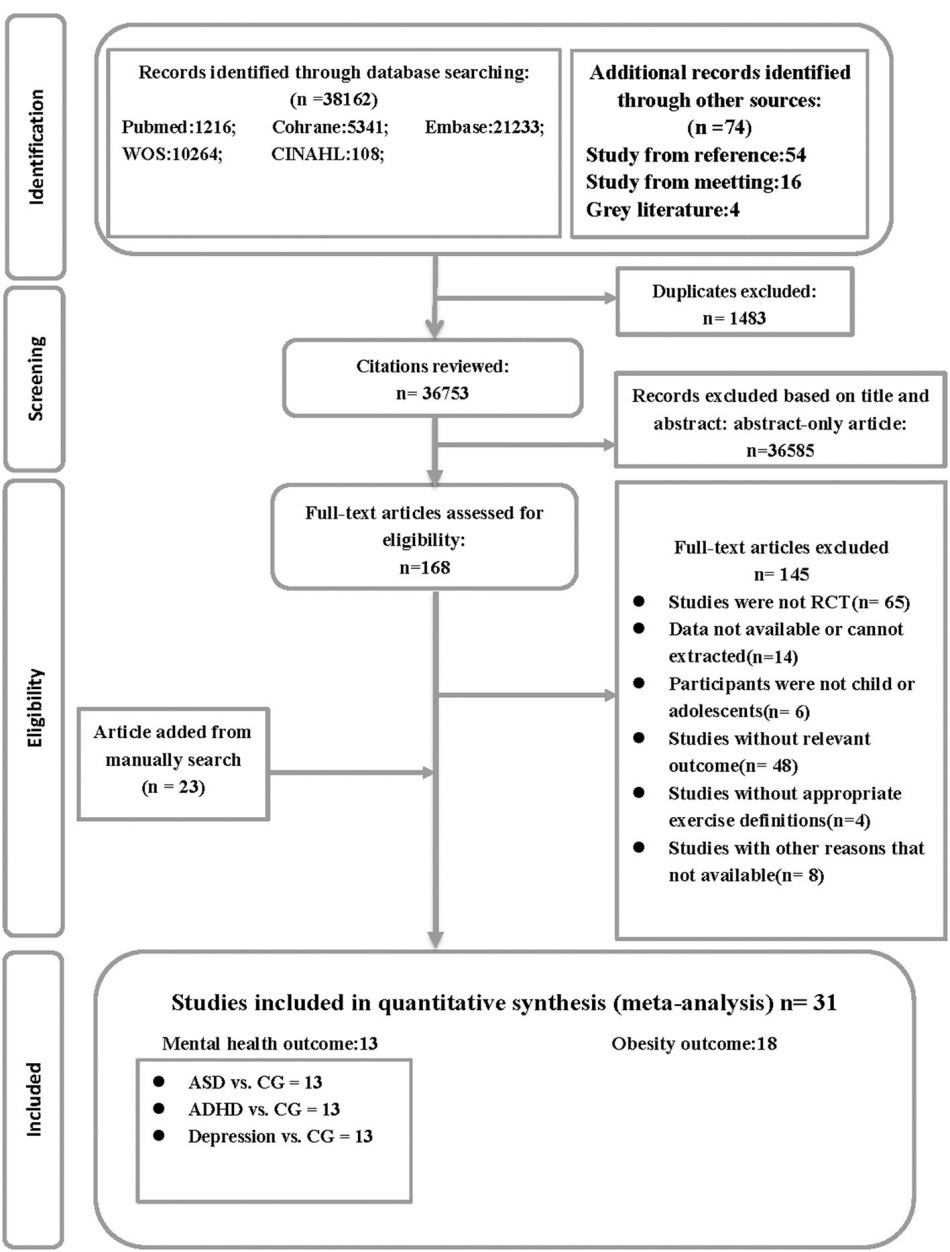
Flow chart. ASD, autism spectrum disorder; ADHD, attention deficit hyperactivity disorder; CG, control group; RCT, randomized controlled trials; WOS, web of science.

Our database search gathered 38,236 citations in addition to 128 studies found by hand search, among which 1,483 studies were removed due to duplication. We excluded 36,585 studies when screening their titles and abstracts, and 145 articles were further excluded on account of various reasons which were outlined in the Flow chart. Eventually, 31 RCTs fulfilled the final criteria on the topic related to children's and adolescents' health. Figure 1 summarizes the detailed process of study selection.

### Characteristics and quality of individual studies

The 31 individual studies, whose publication year ranged from 2004 to 2019 (Hagströmer et al., 1900; Kelly et al., 2004; Kim et al., 2006, 2008; Meyer et al., 2006; Shaibi et al., 2006; Bass et al., 2009; Farpour-Lambert et al., 2009; Karacabey, 2009; Ounis et al., 2010; Mohammadi, 2011; Roshan et al., 2011; Davis et al., 2012; Lee, 2012; Park et al., 2012; Schranz et al., 2012; Seo et al., 2012; Alberga et al., 2013; Hughes et al., 2013; Movahedi et al., 2013; Choi et al., 2014; Lau et al., 2014; Bahrami et al., 2015; Carter et al., 2015; Hay et al., 2015; Borgi et al., 2016; Chen et al., 2016; Memarmoghaddam et al., 2016; Nobre et al., 2017; Pan et al., 2017; Abdelmotaleb et al., 2019) involved 1,255 participants, among whom 646 were active participants and 609 were control subjects. Of the 13 studies (Bass et al., 2009; Mohammadi, 2011; Roshan et al., 2011; Lee, 2012; Hughes et al., 2013; Movahedi et al., 2013; Choi et al., 2014; Bahrami et al., 2015; Carter et al., 2015; Borgi et al., 2016; Memarmoghaddam et al., 2016; Pan et al., 2017; Abdelmotaleb et al., 2019) evaluating neurodevelopment disorders and depression, 4 focus on ASD (Bass et al., 2009; Movahedi et al., 2013; Bahrami et al., 2015; Borgi et al., 2016), 5 on ADHD (Lee, 2012; Choi et al., 2014; Memarmoghaddam et al., 2016; Pan et al., 2017; Abdelmotaleb et al., 2019) and 4 on depression (Mohammadi, 2011; Roshan et al., 2011; Hughes et al., 2013; Carter et al., 2015). The diagnosis of each disorder mentioned above was mainly made according to DSM-IV. Most of the studies recruited from the obese or overweight pediatric population using the measurement of body mass index (BMI) for age and gender according to their local growth criteria. BMI from 85 to 95th percentile was rated as overweight and BMI over 95th percentile as obese. As for the mental health studies, the median intervention duration of the PA was 12 weeks (ranging from 6 to 81 weeks) except for one trial without available data (Lee, 2012), and the median intervention duration for the obesity studies was also 12 weeks (ranging from 6 to 27 weeks). Speaking of the study areas, ninewere conducted in America, six in Europe, 14 in Asia, and the remaining two in Australia and Africa, respectively. All trials launched their intervention of exercise with a group setting, and in most studies, the intervention in the youth population was done in specialized venues rather than in schools. The vast majority of trials were conducted with traditional PA to examine traditional exercise such as running and team sports while some focused on novel treatments such as intermittent walking in water and yoga (Roshan et al., 2011; Seo et al., 2012). Key information for the 31 included studies are presented in Supplementary Tables 1, 2. Random sequence generation was reported at length in all RCTs and the required data were available in 30 studies except one (Mohammadi, 2011) which lacked a few characteristics. Overall ROB in all the studies was rated as low apart from several RCTs with potentially high bias for issues such as allocation concealment, blinding method and other bias (Hagströmer et al., 1900; Lee, 2012; Memarmoghaddam et al., 2016). Details related to the quality appraisal are depicted in Supplementary Figures 1, 2.

### Primary outcome results

#### Mental health

**Figure 2:**
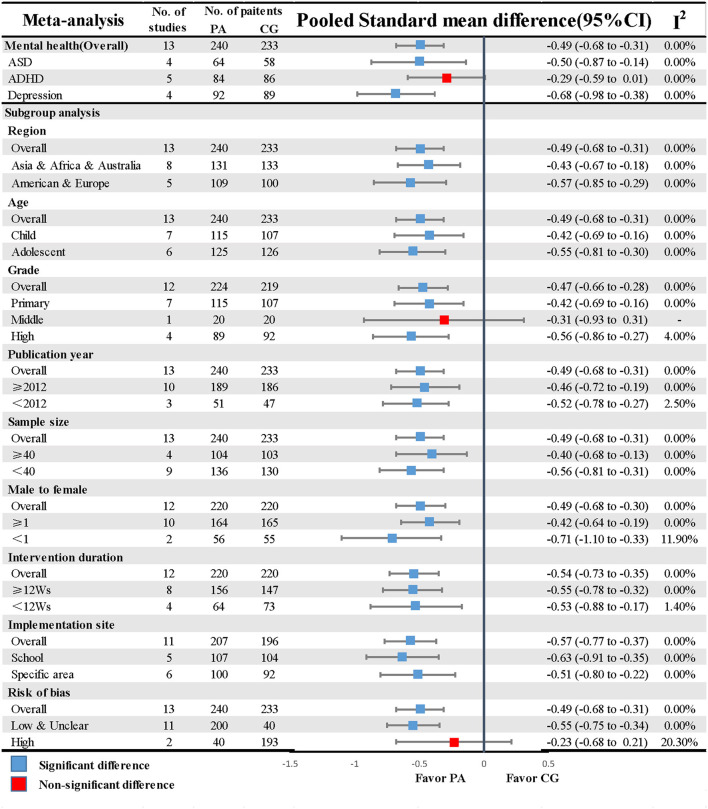
Primary results of the neurodevelopment disorders and depression studies. ASD, autism spectrum disorder; ADHD, attention deficit hyperactivity disorder; CG, control group; CI, confidence interval; PA, physical exercise; Ws, weeks.

Thirteen studies described estimates of the association between PA and neurodevelopment disorders and depression, with three subtype partitions, namely, ASD, ADHD, and depression. PA, compared with the control group, had a significantly positive improvement for ASD (SMD = −0.50, CI: −0.87, −0.14) and depression (SMD = −0.68, CI: −0.98, −0.38) but no significant effect for ADHD (SMD = −0.29, CI: −0.59, 0.01). No significant heterogeneity was found from the whole network of each outcome as their indicator makers, I^2^, were all 0% as shown in Figure 2. A relative symmetry was presented visually by producing a funnel plot (Supplementary Figure 3), and an Egger's test did not detect the existence of publication bias (*P* = 0.69; Supplementary Figure 5).

#### Obesity

**Figure 3:**
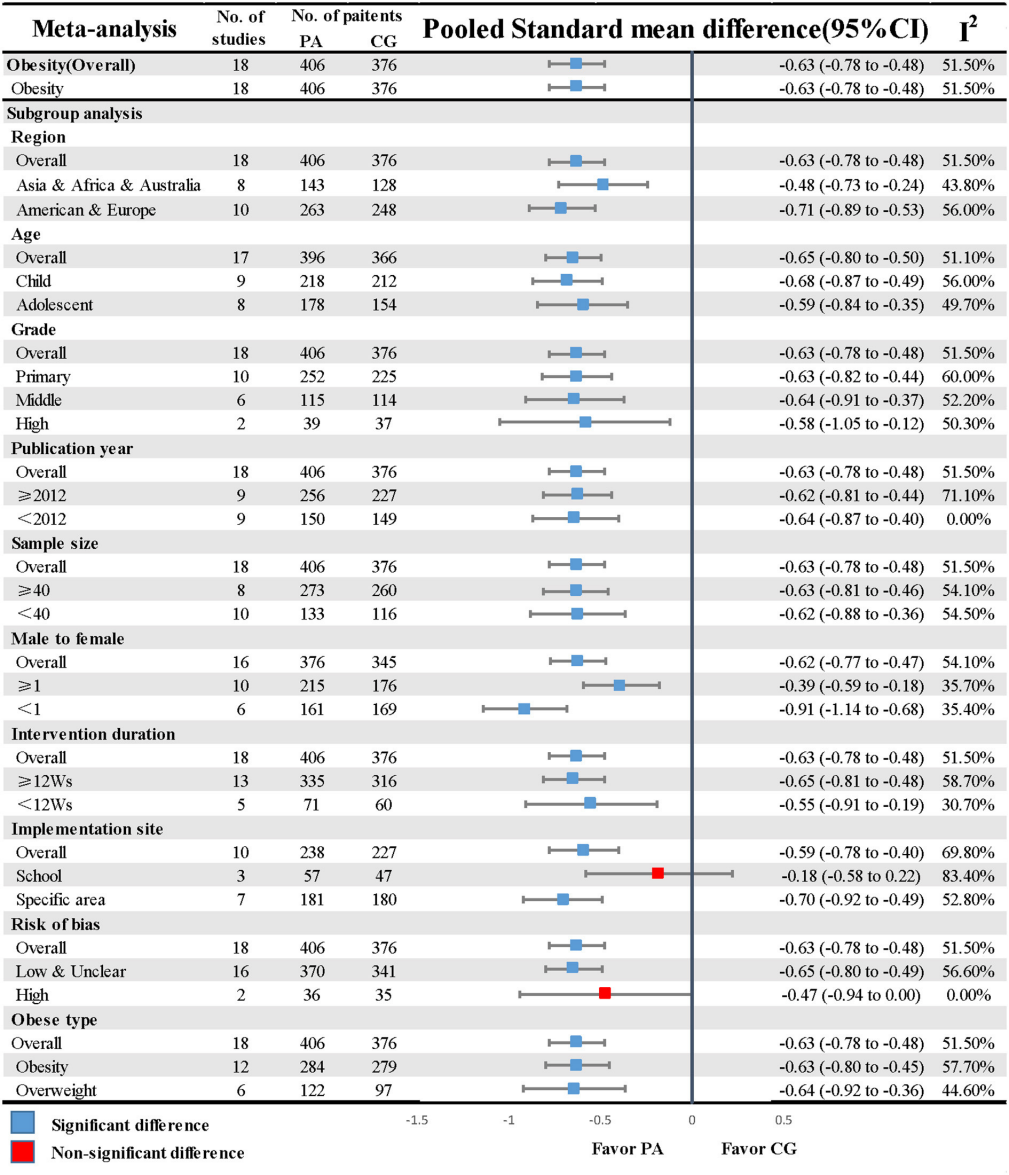
Primary results of the obesity and overweight studies. CG, control group; CI, confidence interval; PA, physical exercise; Ws, weeks.

A total of 18 RCTs evaluated the association between PA and obesity. The PA group (SMD = −0.58, CI: −0.80, −0.36) generated a significant and positive pooled effect size compared with the control group in spite of moderate heterogeneity (*I*^2^ = 55.1%, *P* = 0.006; Figure 3). Although a random model was conducted later, the size of the estimate was still similar to the former one (I^2^ = 49.2%, *P* = 0.01), suggesting there was an obvious heterogeneity. The inspection of a funnel plot showed a slight asymmetry (Supplementary Figure 4), and an Egger's test hinted that potential publication bias might exist (*P* = 0.02; Supplementary Figure 6).

#### Subgroup analyses

We explored the difference between the items of study and the potential sources of heterogeneity by implementing subgroup analysis stratified by region, age, and grade while the remaining subgroups were categorized by publication year, total sample size, male-to-female ratio, intervention duration, implementation site, and study quality. The obesity outcome was further classified into two types (obese and overweight). Based on mental health outcomes, an overwhelming majority of these analyses yielded consistent findings that the SMDs of different groups in the seven subgroup analyses revealed no difference when sub-items were compared with each other; the same result was also found in the comparison between the obesity and overweight groups. Conversely, we observed some differences in study quality [Mental health low and unclear ROB (SMD = −0.55, CI: −0.75, −0.34) vs. high ROB (SMD = −0.23, CI: −0.68, 0.21)] and implementation site [Obesity: conduct in school (SMD = −0.18, CI: −0.58, 0.22) vs. conduct in specialized venues (SMD = −0.70, CI: −0.92, −0.49)]. The results of subgroup analyses based on the 10 pre-specified covariates are listed in Figures 2, 3.

## Discussion

In order to address the ongoing controversy about whether PA is truly effective in the improvement of neurodevelopment disorders, depression, and obesity among children and adolescents, our research identified 31 RCTs consisting of 1,255 children and adolescents. The results suggested that participants receiving the PA intervention showed significant improvement in ASD, depression, and obesity compared with the control group, but no significant effect on ADHD was observed, which was nearly consistent with the current meta-analyses. Therefore, the above findings indicate that more appropriate physical activities should be recommended among the youth population. Moreover, teachers and parents should ensure that children engage themselves in a moderate-to-vigorous physical activity program either in the school or at home and, if possible, engage in PA in group settings.

What should be highlighted prior to a comprehensive discussion of our main findings is that PA is beneficial for mitigating symptoms in children and adolescents or improving their wellbeing rather than curing the disorder completely. For example, on the basis of various aerobic-based interventions, we concluded that children with mental problems obtained more significant improvement for symptoms such as executive function, language skill, or social interaction ability in the active group than in the control group. Furthermore, considering that the multi-incertitude natural trajectory over time would change as children grow, our implications should be interpreted in the context of other multiple modifiable predictors of children' health.

Our main finding was consistent with the results in previous literature-based studies where PA, on account of its effectiveness alongside medical treatment, was recommended for overall health management in children and adolescents (Oliveira et al., 2016; Healy et al., 2018; Rodriguez-Ayllon et al., 2019), as well as for adults diagnosed with diseases but who were unresponsive to medication, or for patients seeking for alternative treatments (Ashton et al., 2020; Garcia-Hermoso et al., 2020). A prior meta-analysis regarding the association between PA and patients with ASD included eleven trials whose analyses were pooled with different types of trials (RCT, quasi-experimental trial, and quasi-control trial), which did not comply with the instructions of Cochrane, and in this study, no significant difference was revealed in the association between PA and children with ASD. Compared with the previous meta-analysis performed by Howells et al. (2019) an extensive literature search was conducted by using electronic databases and hand searching conference abstracts for relevant literature (Howells et al., 2019). Although only 4 studies were included to evaluate the relationship between PA and ASD, all of them were RCTs which makes our findings more reliable. In fact, results from a collection of studies imply that a PA intervention is potentially more suitable for adults than for youth as it is originally intended for the former (Verburgh et al., 2014). As has been mentioned, previous trials described that PA demonstrates its effect not only on different populations but also on various diseases such as dementia, diabetes, and Parkinson's (Goodwin et al., 2008; Liang et al., 2019; Wu et al., 2019). Despite the fact that our results revealed no significant relationship between PA and ADHD, a similar study drew the conclusion that PA had significantly improved a variety of behaviors and social problems among children affected by ADHD (Zang, 2019). The inconsistency in results possibly arises from study design since different types of studies were included in the two meta-analyses, as well as criteria factors such as inclusion criteria and outcome selection, which were also considered to have contributed to the differences. Neurodiversity is worth mentioning here and is often used synonymously with neurodivergence to describe the brain's wide range of normal variation, experience, and interaction with the world as having no “right” way. Neurodiversity views conditions like autism, ADHD, and dyslexia as differences rather than deficits. These differences may present challenges to functioning in a predominantly “neurotypical” society, but they are not innate. Neurodiversity in medicine is mostly studied in small, qualitative studies and focuses mainly on autism (Taylor, 2021). In addition to fostering healthy growth and development, physical activity can make youths feel better, function better, sleep better, and reduce chronic disease risks. As a result, providing young people with opportunities and encouraging them to engage in various and enjoyable physical activities that are appropriate for their age is of crucial importance (Piercy et al., 2018). Further studies are needed to explain the neurobiological and behavioral mechanisms that elicit the beneficial impact of PA on mental health among youth.

The multiple mechanisms of the positive relationship between PA and obesity in youth are well-established (Kelly et al., 2004; Farpour-Lambert et al., 2009), but the evidence for this association on mental health is still debated. In this context, we note that a series of neurobiological, psychosocial, and behavioral mechanisms have been proved previously (Lubans et al., 2016). A tentative explanation of a plausible underlying effect is that exercise may be favorable for creating a causal link between physical self-concept and mental health (Lubans et al., 2016; Rodriguez-Ayllon et al., 2019), or contribute a positive impact on the structure and function of one's brain which may directly improve their mental health (Hillman et al., 2014). However, there is insufficient reliable evidence to draw a true conclusion regarding any of these existing mechanisms. Despite these promising findings, researchers have not yet developed a compelling theoretical framework for interpreting exercise-based interventions' positive effects on mental health in the youth population. Some studies, however, explain that these benefits may be linked to neurobiological mechanisms in light of recent discoveries in psychology and neuroscience. Exercise has been shown to increase levels of brain-derived neurotrophic factor (Carl et al., 2002; Ang and Gomez-Pinilla, 2007; Langdon and Corbett, 2012; BDNF). There are several important functions of BDNF, including neurological function, neurogenesis, and regulating the survival and differentiation of neurons, as well as guiding the path of axonal growth and synaptic plasticity (Phillips et al., 2014). There has been an association between BDNF levels in serum and symptom severity in children with autism spectrum disorders (Kasarpalkar et al., 2014). BDNF has been linked to exercise-associated cognitive enhancement in multiple research studies, including (1) BDNF is upregulated for a period of up to 7 days after exercise (Berchtold et al., 2005), (2) BDNF receptors appear to be required for exercise-induced cognitive enhancement (Langdon and Corbett, 2012), (3) but blocking them reduces these benefits (Vaynman et al., 2006; Ang and Gomez-Pinilla, 2007). BDNF, exercise, and psychiatric symptoms have been correlative, which suggests that exercise-based interventions increase neural plasticity and youth mental health has improved, which is reflected in their inherent abilities including communication, learning, and language. Compared to seated rest, participants were more motivated to complete mental work after exercise. Despite the fact that abnormal motivation is thought to be a key element of ADHD, motivation to complete mental work is rarely measured in studies on exercise effects. After 20 min of moderate-intensity exercise, the motivation to complete cognitive tasks significantly increased. There has been evidence that ADHD patients have a reduced availability of D2/D3 receptors in the nucleus accumbens, an area in the striatum associated with motivation (Volkow et al., 2011). There are also several points indicating that the beneficial effect of PA may be due to the changes in eye-blink responses (Wigal et al., 2003) and reductions in motor impersistence (Tantillo et al., 2002) are associated with the improvement of hippocampal long-term potentiation (Praag et al., 1999a), neurogenesis (Praag et al., 1999b), and mRNA expression of hippocampal and neocortical neurotrophins (Neeper et al., 1991).

The results from the subgroup analysis displayed a significant difference between several items. The risk of bias in our outcomes revealed a non-significant difference in high ROB compared with low and unclear bias. In addition, we observed that children who received interventions in specific areas experienced a significant effect compared with those at school, whose pooled effect sizes were non-significant. Determining the basis of group interaction via subgroup analysis helped us understand the inherent association between them and provided far-reaching implications for the direction of future studies, especially for large population-based RCTs.

It is worth mentioning that existing meta-analyses have evaluated the efficacy of diet alone, diet combined with exercise, or exercise only for overweight children, and have indicated that exercise in conjunction with diet-focused components seemed to be more effective in reducing metabolic risks (Ho et al., 2013). However, whether adding other programs such as sleeping patterns or positive emotions to exercise-based interventions would be truly more effective than the administration of PA alone is unclear. Aerobic-based interventions with moderate effect size and launched in group settings are probably promising treatments for physical and mental problems, obesity in particular. Notably, there are several types of PA which were divided into three categories, namely, aerobic exercise, resistance exercise, and mind-body exercise, which was a broad cross-disciplinary recommendation across psychology and allied health professions (Zou et al., 2019), but rarely used for youth. A large majority of the included studies implemented aerobic exercise and resistance exercise, and one explored the effect of mind-body exercise with yoga training (Seo et al., 2012). Although the current evidence was rather inconclusive on which type of PA was the optimal exercise to improve children's health, it was pointed out that more marked benefits of aerobic exercise for youth would be attained with multi-component activities, such as team sports, karate techniques training, and conventional exercise such as fast walking, running and swimming (Ounis et al., 2010; André and Béguier, 2015; Chen et al., 2016). Based on sports epidemiology, the compositional isotemporal substitution model (CISM), namely, an approach for evaluating changes with healthy outcome by reallocating time between PA and sedentary behavior (SB) (Mekary et al., 2009), also deserves a mention. Previous studies on CISM have found a beneficial impact on mental health outcomes (Janssen, 2016), body fat (Aggio et al., 2015), and body composition (Collings et al., 2017) for replacing total SB with light-intensity PA or moderate-to-vigorous intensity PA among youth individuals.

For neurodevelopment disorders and depression outcomes, we excluded the universal-based prevention and treatment RCTs since such studies might synergistically enhance the effect size of PA, which would be unfavorable for our final analysis. For example, universal prevention is aimed at participants who have no depression symptoms, while in treatment studies, participants experience a high level of depression or are diagnosed with depression. Children's public health, an issue highly valued by national governments, largely and indirectly affects the enhancement of national quality. Many kinesiologists hold the view that children maintain a healthy body through moderate daily exercise. However, there is little consensus on what exercise is required for youth, how many times they need to participate in the target program, or which age-adapted versions of PA should be used in which specific stage of youth. It is extremely complicated for professionals to decide whether health issues occurring in this age period are developmental or mental. The aforementioned debate can be settled by conducting a series of high-quality studies with a set of standardized tasks performed in any type of PA involving a large number of populations.

Our results need cautious interpretation since several limitations were noteworthy. First, heterogeneity was found in the obesity outcomes, which may be attributed to the study designs and methodological differences between studies. Second, the quality of several studies potentially threatened the reliability of our main findings. Furthermore, a relatively narrow range of studies implemented blinded studies to children and treatment personnel during the process of intervention, causing an inherent risk of performance bias.

## Conclusion

In conclusion, our preliminary finding is that PA has a moderate beneficial effect in improving the symptoms of ASD, depression, and obesity in children and adolescents. The currently available evidence is unable to support the conclusion that PA has a significant improvement for children with ADHD. To supply more evidence for the generalization of the conclusion, further multicenter studies should preferably perform high-quality RCTs with large population-based PA interventions.

## Data availability statement

The original contributions presented in the study are included in the article/Supplementary material, further inquiries can be directed to the corresponding author.

## Author contributions

SP conducted the database search, screened and extracted data for the meta-analysis, prepared extracted data for the procedures, and had primary responsibility in writing this article. SP, YF, and AO performed statistical analysis and interpretation of data. SP and JL contributed to the discussion and editing. JL critically revised the draft manuscript. All authors read and approved the final manuscript.

## Funding

This research was supported by the Fundamental Research Funds for the Central Universities (Hohai University, B200202203).

## Conflict of interest

The authors declare that the research was conducted in the absence of any commercial or financial relationships that could be construed as a potential conflict of interest.

## Publisher's note

All claims expressed in this article are solely those of the authors and do not necessarily represent those of their affiliated organizations, or those of the publisher, the editors and the reviewers. Any product that may be evaluated in this article, or claim that may be made by its manufacturer, is not guaranteed or endorsed by the publisher.

## Supplementary material

Supplementary Figure 1 Risk of bias graph.

Supplementary Figure 2 Risk of bias summary.

Supplementary Figure 3 Funnel plot based on the neurodevelopment disorders and depression outcomes.

Supplementary Figure 4 Funnel plot based on the obesity outcome.

Supplementary Figure 5 Egger's test based on the neurodevelopment disorders and depression outcomes.

Supplementary Figure 6 Egger's test based on the obesity outcome.

Supplementary Table 1 Demographic characteristics of the included studies and their participants.

Supplementary Table 2 The demographic characteristics of included studies.

Supplementary Table 3 PRISMA 2020 checklist.
