# Supplementary figures and images for: Meta-analysis and systematic review of physical activity on neurodevelopment disorders, depression, and obesity among children and adolescents
Source: Front Psychol. 2022 Nov 30;13:940977. doi: 10.3389/fpsyg.2022.940977 (PMC9747947; doi:10.3389/fpsyg.2022.940977)

**Supplement Figure.1 Risk of Bias graph**

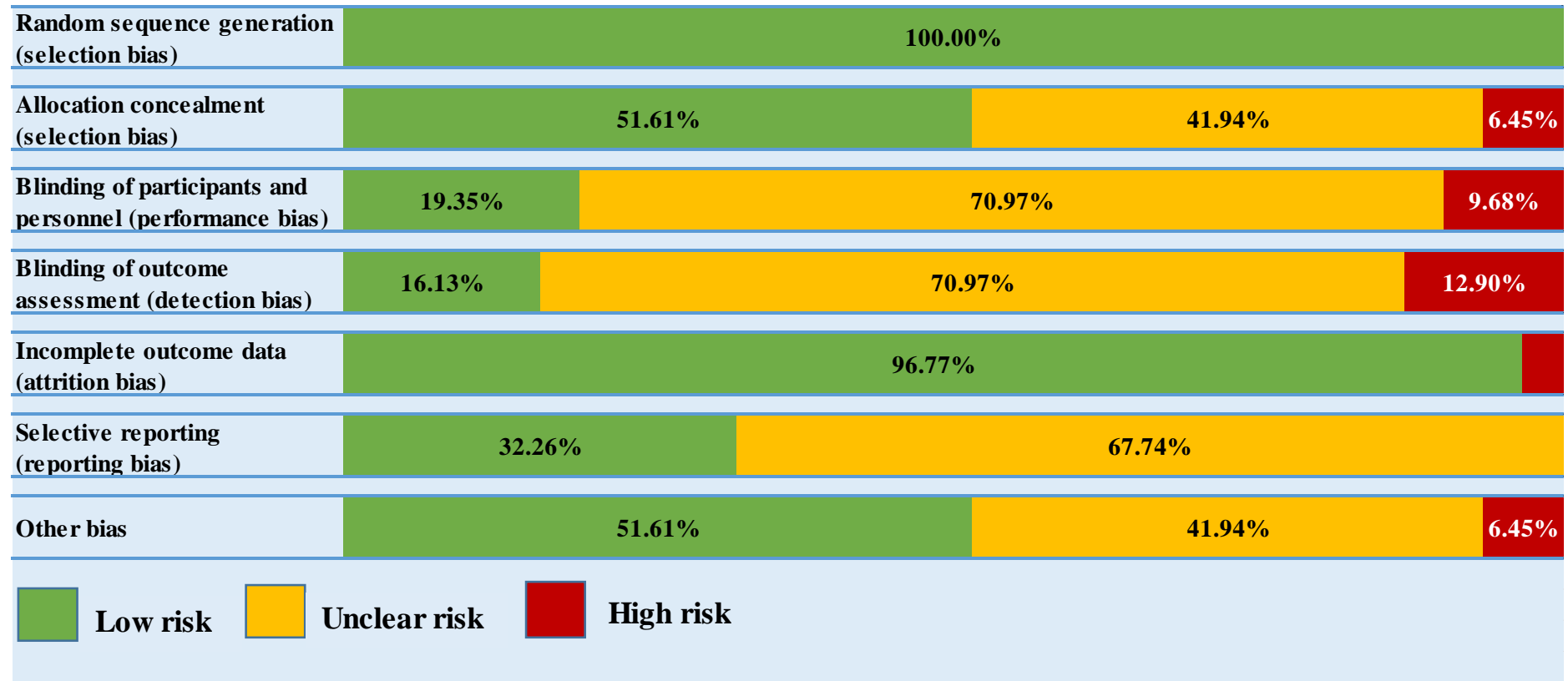

Supplement: Supplementary Figure 1 — Risk of bias graph. [file Data_Sheet_1.PDF]

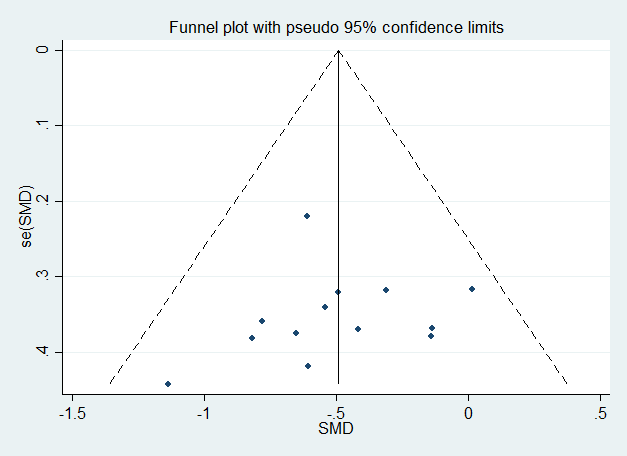

Supplement: Supplementary Figure 3 — Funnel plot based on the neurodevelopment disorders and depression outcomes. [file Image_1.TIF]

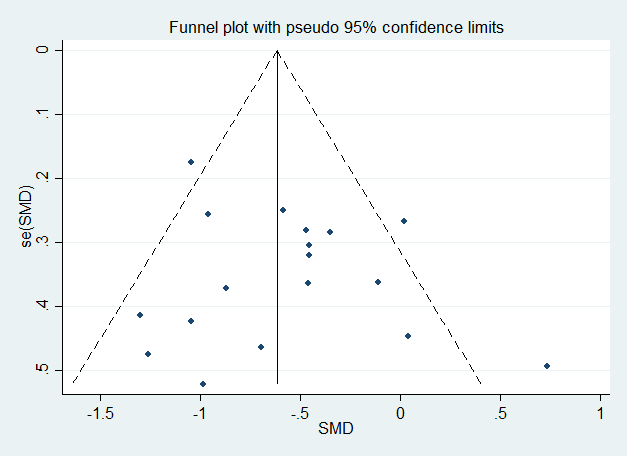

Supplement: Supplementary Figure 4 — Funnel plot based on the obesity outcome. [file Image_2.TIF]

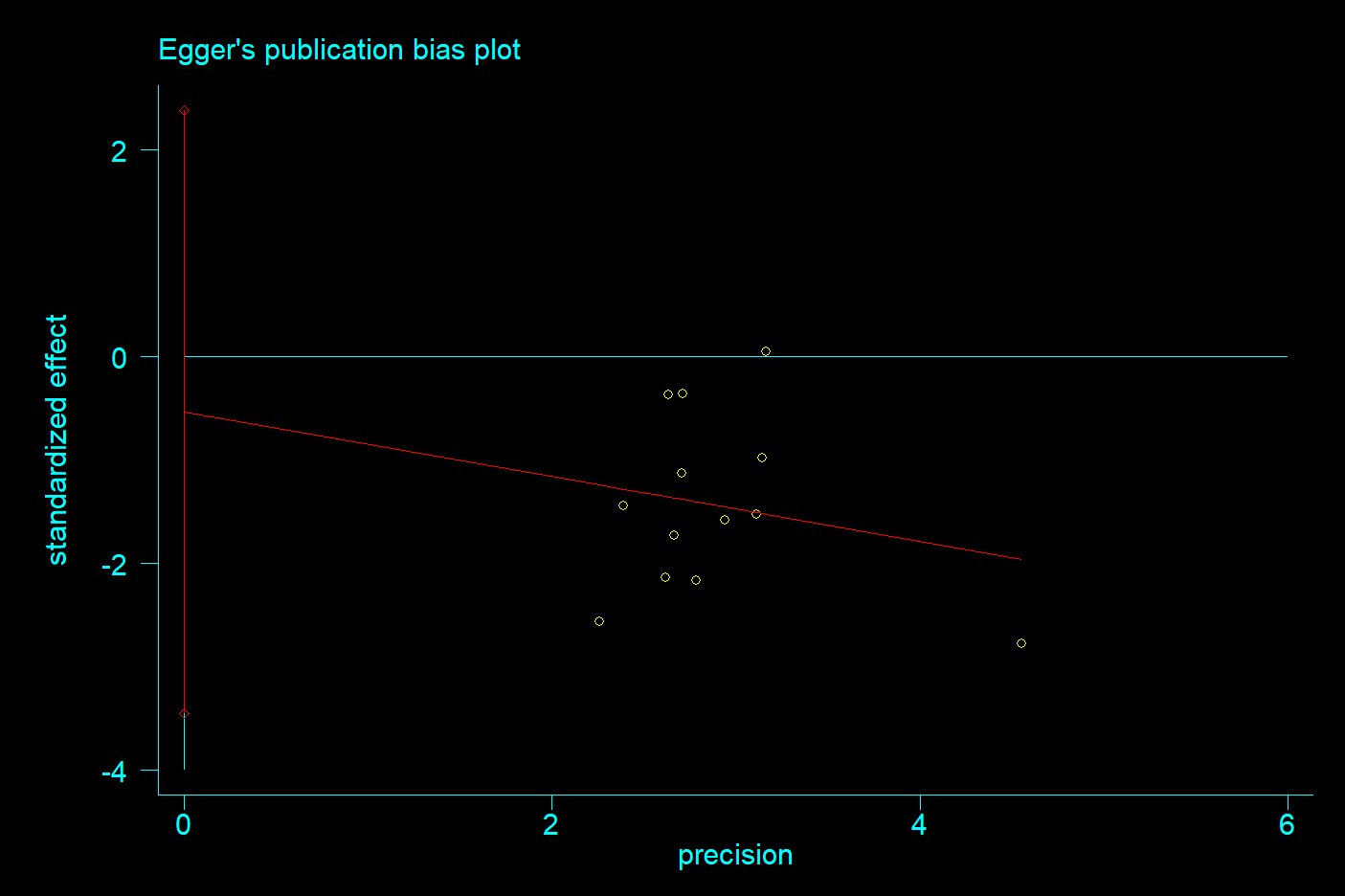

Supplement: Supplementary Figure 5 — Egger's test based on the neurodevelopment disorders and depression outcomes. [file Image_3.TIF]

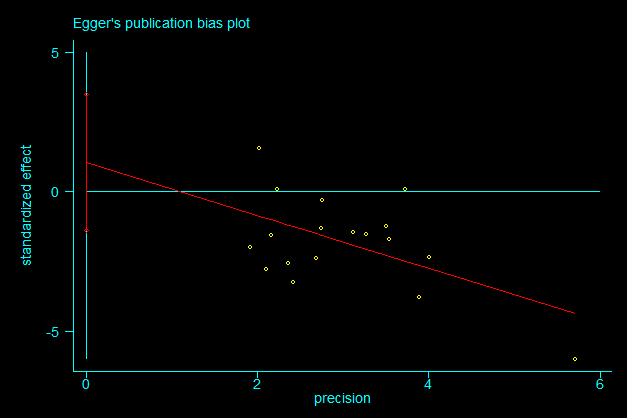

Supplement: Supplementary Figure 6 — Egger's test based on the obesity outcome. [file Image_4.TIF]
