# Supplementary material for: Meta-analysis and systematic review of physical activity on neurodevelopment disorders, depression, and obesity among children and adolescents
Source: Front Psychol. 2022 Nov 30;13:940977. doi: 10.3389/fpsyg.2022.940977 (PMC9747947; doi:10.3389/fpsyg.2022.940977)

Supplement Figure.2 Risk of Bias Summary

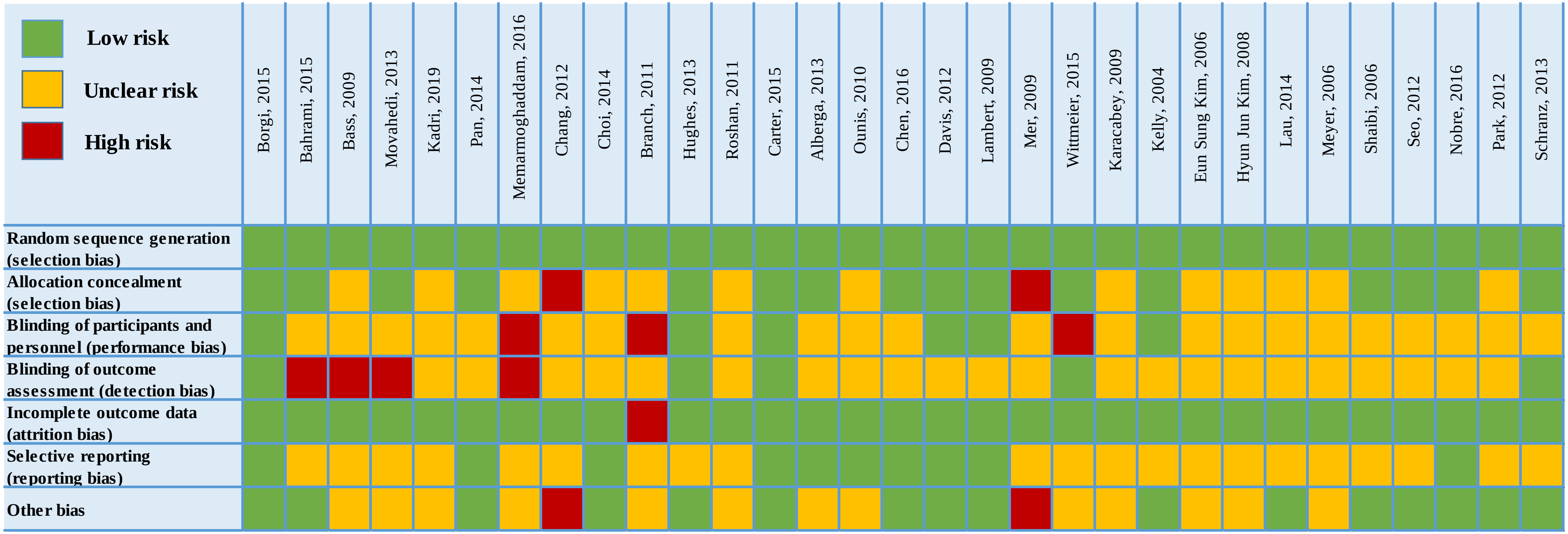

Supplement: Supplementary Figure 2 — Risk of bias summary. [file Data_Sheet_2.PDF]
