# Supplementary material for: Meta-analysis and systematic review of physical activity on neurodevelopment disorders, depression, and obesity among children and adolescents
Source: Front Psychol. 2022 Nov 30;13:940977. doi: 10.3389/fpsyg.2022.940977 (PMC9747947; doi:10.3389/fpsyg.2022.940977)
Supplement: Supplementary file 3 [file Data_Sheet_3.PDF]

PubMed search strategy:

#1 Search (“Students”[Mesh] public health)

#2 Search (Student [Title/Abstract]) OR School Enrollment [Title/Abstract]) OR (Enrollments, School [Title/Abstract]) OR (School Enrollments [Title/Abstract])

#3 Search (“Child”[Mesh])

#4 Search (Children [Title/Abstract])

#5 Search (“Adolescent”[Mesh])

#6 Search (Adolescents [Title/Abstract]) OR (Adolescence [Title/Abstract]) OR (Teens [Title/Abstract]) OR (Teenagers [Title/Abstract]) OR (Youths [Title/Abstract]) OR (Adolescents, Female [Title/Abstract]) OR (Female Adolescent [Title/Abstract]) OR (Male Adolescents [Title/Abstract])

#7 Search (“Students”[Mesh]) OR (Student [Title/Abstract]) OR School Enrollment [Title/Abstract]) OR (Enrollments, School [Title/Abstract]) OR (School Enrollments [Title/Abstract]) OR (“Child”[Mesh]) OR (Children [Title/Abstract]) OR (“Adolescent”[Mesh]) OR (Adolescents [Title/Abstract]) OR (Adolescence [Title/Abstract]) OR (Teens [Title/Abstract]) OR (Teenagers [Title/Abstract]) OR (Youths [Title/Abstract]) OR (Adolescents, Female [Title/Abstract]) OR (Female Adolescent [Title/Abstract]) OR (Male Adolescents [Title/Abstract])

#8 Search (“Mental health”[Mesh])

#9 Search (Health, Mental) [Title/Abstract] OR (Mental Hygiene [Title/Abstract]) OR (Hygiene, Mental [Title/Abstract]) OR (Nursing, Mental Health [Title/Abstract]) OR (Nursing, Psychiatric [Title/Abstract]) OR (Psychosocial Nursing [Title/Abstract]) OR (Rehabilitation, Psychiatric [Title/Abstract]) OR (Mental Health Rehabilitation [Title/Abstract]) OR (Rehabilitation, Mental Health[Title/Abstract]) OR (Rehabilitation, Psychosocial [Title/Abstract])

#10 Search (“Pediatric Obesity”[Mesh])

#11 Search (Obesity, Pediatric) [Title/Abstract] OR (Childhood Onset Obesity [Title/Abstract]) OR (Obesity, Childhood Onset [Title/Abstract]) OR (Obesity in Childhood [Title/Abstract]) OR (Child Obesity [Title/Abstract]) OR (Childhood Obesity [Title/Abstract]) OR (Obesity, Childhood [Title/Abstract]) OR (Infant Overweight [Title/Abstract]) OR (Infantile Obesity[Title/Abstract]) OR (Infant Obesity [Title/Abstract]) OR (Childhood Overweight [Title/Abstract]) OR (Adolescent Overweight [Title/Abstract]) OR (Adolescent Obesity[Title/Abstract]) OR (Obesity in Adolescence [Title/Abstract])

#12 Search (“Exercises”[Mesh])

#13 Search (Physical Activity [Title/Abstract]) OR (Activities, Physical [Title/Abstract]) OR (Physical Activities [Title/Abstract]) OR (Exercise, Physical [Title/Abstract]) OR (Exercises, Physical [Title/Abstract]) OR (Physical Exercise [Title/Abstract]) OR (Physical Exercises [Title/Abstract]) OR (Acute Exercise [Title/Abstract]) OR (Acute Exercises [Title/Abstract]) OR

(Exercise, Acute [Title/Abstract]) OR (Exercises, Acute [Title/Abstract]) OR (Exercise, Isometric [Title/Abstract]) OR (Exercises, Isometric [Title/Abstract]) OR (Isometric Exercises[Title/Abstract]) OR (Exercise, Aerobic [Title/Abstract]) OR (Aerobic Exercise [Title/Abstract]) OR (Exercises, Aerobic [Title/Abstract]) OR (Exercise Trainings [Title/Abstract]) OR (Training, Exercise [Title/Abstract]) OR (Trainings, Exercise [Title/Abstract])

#14 Search (Randomized Controlled Trial [Title/Abstract]) OR (Randomized [Title/Abstract]) OR (Randomized Controlled Trials [Title/Abstract]) OR (Randomised Controlled Trials [Title/Abstract]) OR (RCT [Title/Abstract]) OR (RCTs [Title/Abstract])

#15 Search ("Students"[Mesh]) OR (Student [Title/Abstract]) OR School Enrollment[Title/Abstract]) OR (Enrollments, School [Title/Abstract]) OR (School Enrollments [Title/Abstract]) OR ("Child"[Mesh]) OR (Children [Title/Abstract]) OR Search ("Adolescent"[Mesh]) OR Search (Adolescents [Title/Abstract]) OR (Adolescence [Title/Abstract]) OR (Teens [Title/Abstract]) OR (Teenagers [Title/Abstract]) OR (Youths [Title/Abstract]) OR (Adolescents, Female [Title/Abstract]) OR (Female Adolescent [Title/Abstract]) OR (Male Adolescents [Title/Abstract]) OR ("Students"[Mesh]) OR (Student [Title/Abstract]) OR School Enrollment[Title/Abstract]) OR (Enrollments, School [Title/Abstract]) OR (School Enrollments [Title/Abstract]) OR ("Child"[Mesh]) OR (Children [Title/Abstract]) OR ("Adolescent"[Mesh]) OR (Adolescents [Title/Abstract]) OR (Adolescence [Title/Abstract]) OR (Teens [Title/Abstract]) OR (Teenagers [Title/Abstract]) OR (Youths [Title/Abstract]) OR (Adolescents, Female [Title/Abstract]) OR (Female Adolescent [Title/Abstract]) OR (Male Adolescents [Title/Abstract])

#16 Search ("Mental health"[Mesh]) OR (Health, Mental) [Title/Abstract] OR (Mental Hygiene [Title/Abstract]) OR (Hygiene, Mental [Title/Abstract]) OR (Nursing, Mental Health [Title/Abstract]) OR (Nursing, Psychiatric [Title/Abstract]) OR (Psychosocial Nursing [Title/Abstract]) OR (Rehabilitation, Psychiatric [Title/Abstract]) OR (Mental Health Rehabilitation [Title/Abstract]) OR (Rehabilitation, Mental Health[Title/Abstract]) OR (Rehabilitation, Psychosocial [Title/Abstract])

#17 Search ("Pediatric Obesity"[Mesh]) OR (Obesity, Pediatric) [Title/Abstract] OR (Childhood Onset Obesity [Title/Abstract]) OR (Obesity, Childhood Onset [Title/Abstract]) OR (Obesity in Childhood [Title/Abstract]) OR (Child Obesity [Title/Abstract]) OR (Childhood Obesity [Title/Abstract]) OR (Obesity, Childhood [Title/Abstract]) OR (Infant Overweight [Title/Abstract]) OR (Infantile Obesity[Title/Abstract]) OR (Infant Obesity [Title/Abstract]) OR (Childhood Overweight [Title/Abstract]) OR (Adolescent Overweight [Title/Abstract]) OR (Adolescent Obesity[Title/Abstract]) OR (Obesity in Adolescence [Title/Abstract])

#18 Search ("Exercises"[Mesh]) OR (Physical Activity [Title/Abstract]) OR (Activities, Physical [Title/Abstract]) OR (Physical Activities [Title/Abstract]) OR (Exercise, Physical [Title/Abstract]) OR (Exercises, Physical

[Title/Abstract]) OR (Physical Exercise [Title/Abstract]) OR (Physical Exercises [Title/Abstract]) OR (Acute Exercise [Title/Abstract]) OR (Acute Exercises [Title/Abstract]) OR (Exercise, Acute [Title/Abstract]) OR (Exercises, Acute [Title/Abstract]) OR (Exercise, Isometric [Title/Abstract]) OR (Exercises, Isometric [Title/Abstract]) OR (Isometric Exercises[Title/Abstract]) OR (Exercise, Aerobic [Title/Abstract]) OR (Aerobic Exercise [Title/Abstract]) OR (Exercises, Aerobic [Title/Abstract]) OR (Exercise Trainings [Title/Abstract]) OR (Training, Exercise [Title/Abstract]) OR (Trainings, Exercise [Title/Abstract])

#19 Search (“Students”[Mesh]) OR (Student [Title/Abstract]) OR School Enrollment[Title/Abstract]) OR (Enrollments, School [Title/Abstract]) OR (School Enrollments [Title/Abstract]) OR (“Child”[Mesh]) OR (Children [Title/Abstract]) OR Search (“Adolescent”[Mesh]) OR Search (Adolescents [Title/Abstract]) OR (Adolescence [Title/Abstract]) OR (Teens [Title/Abstract]) OR (Teenagers [Title/Abstract]) OR (Youths [Title/Abstract]) OR (Adolescents, Female [Title/Abstract]) OR (Female Adolescent [Title/Abstract]) OR (Male Adolescents [Title/Abstract]) OR (“Students”[Mesh]) OR (Student [Title/Abstract]) OR School Enrollment[Title/Abstract]) OR (Enrollments, School [Title/Abstract]) OR (School Enrollments [Title/Abstract]) OR (“Child”[Mesh]) OR (Children [Title/Abstract]) OR (“Adolescent”[Mesh]) OR (Adolescents [Title/Abstract]) OR (Adolescence [Title/Abstract]) OR (Teens [Title/Abstract]) OR (Teenagers [Title/Abstract]) OR (Youths [Title/Abstract]) OR (Adolescents, Female [Title/Abstract]) OR (Female Adolescent [Title/Abstract]) OR (Male Adolescents [Title/Abstract]) AND (“Mental health”[Mesh]) OR (Health, Mental) [Title/Abstract] OR (Mental Hygiene [Title/Abstract]) OR (Hygiene, Mental [Title/Abstract]) OR (Nursing, Mental Health [Title/Abstract]) OR (Nursing, Psychiatric [Title/Abstract]) OR (Psychosocial Nursing [Title/Abstract]) OR (Rehabilitation, Psychiatric [Title/Abstract]) OR (Mental Health Rehabilitation [Title/Abstract]) OR (Rehabilitation, Mental Health[Title/Abstract]) OR (Rehabilitation, Psychosocial [Title/Abstract]) AND (“Exercises”[Mesh]) OR (Physical Activity [Title/Abstract]) OR (Activities, Physical [Title/Abstract]) OR (Physical Activities [Title/Abstract]) OR (Exercise, Physical [Title/Abstract]) OR (Exercises, Physical [Title/Abstract]) OR (Physical Exercise [Title/Abstract]) OR (Physical Exercises [Title/Abstract]) OR (Acute Exercise [Title/Abstract]) OR (Acute Exercises [Title/Abstract]) OR (Exercise, Acute [Title/Abstract]) OR (Exercises, Acute [Title/Abstract]) OR (Exercise, Isometric [Title/Abstract]) OR (Exercises, Isometric [Title/Abstract]) OR (Isometric Exercises[Title/Abstract]) OR (Exercise, Aerobic [Title/Abstract]) OR (Aerobic Exercise [Title/Abstract]) OR (Exercises, Aerobic [Title/Abstract]) OR (Exercise Trainings [Title/Abstract]) OR (Training, Exercise [Title/Abstract]) OR (Trainings, Exercise [Title/Abstract]) AND (Randomized Controlled Trial [Title/Abstract]) OR (Randomized [Title/Abstract]) OR (Randomized Controlled Trials [Title/Abstract]) OR (Randomised Controlled

Trials [Title/Abstract]) OR (RCT [Title/Abstract]) OR (RCTs [Title/Abstract])

#20 Search (“Students”[Mesh]) OR (Student [Title/Abstract]) OR School Enrollment[Title/Abstract]) OR (Enrollments, School [Title/Abstract]) OR (School Enrollments [Title/Abstract]) OR (“Child”[Mesh]) OR (Children [Title/Abstract]) OR Search (“Adolescent”[Mesh]) OR Search (Adolescents [Title/Abstract]) OR (Adolescence [Title/Abstract]) OR (Teens [Title/Abstract]) OR (Teenagers [Title/Abstract]) OR (Youths [Title/Abstract]) OR (Adolescents, Female [Title/Abstract]) OR (Female Adolescent [Title/Abstract]) OR (Male Adolescents [Title/Abstract]) OR (“Students”[Mesh]) OR (Student [Title/Abstract]) OR School Enrollment[Title/Abstract]) OR (Enrollments, School [Title/Abstract]) OR (School Enrollments [Title/Abstract]) OR (“Child”[Mesh]) OR (Children [Title/Abstract]) OR (“Adolescent”[Mesh]) OR (Adolescents [Title/Abstract]) OR (Adolescence [Title/Abstract]) OR (Teens [Title/Abstract]) OR (Teenagers [Title/Abstract]) OR (Youths [Title/Abstract]) OR (Adolescents, Female [Title/Abstract]) OR (Female Adolescent [Title/Abstract]) OR (Male Adolescents [Title/Abstract]) AND (“Pediatric Obesity”[Mesh]) OR (Obesity, Pediatric) [Title/Abstract] OR (Childhood Onset Obesity [Title/Abstract]) OR (Obesity, Childhood Onset [Title/Abstract]) OR (Obesity in Childhood [Title/Abstract]) OR (Child Obesity [Title/Abstract]) OR (Childhood Obesity [Title/Abstract]) OR (Obesity, Childhood [Title/Abstract]) OR (Infant Overweight [Title/Abstract]) OR (Infantile Obesity[Title/Abstract]) OR (Infant Obesity [Title/Abstract]) OR (Childhood Overweight [Title/Abstract]) OR (Adolescent Overweight [Title/Abstract]) OR (Adolescent Obesity[Title/Abstract]) OR (Obesity in Adolescence [Title/Abstract]) AND (“Exercises”[Mesh]) OR (Physical Activity [Title/Abstract]) OR (Activities, Physical [Title/Abstract]) OR (Physical Activities [Title/Abstract]) OR (Exercise, Physical [Title/Abstract]) OR (Exercises, Physical [Title/Abstract]) OR (Physical Exercise [Title/Abstract]) OR (Physical Exercises [Title/Abstract]) OR (Acute Exercise [Title/Abstract]) OR (Acute Exercises [Title/Abstract]) OR (Exercise, Acute [Title/Abstract]) OR (Exercises, Acute [Title/Abstract]) OR (Exercise, Isometric [Title/Abstract]) OR (Exercises, Isometric [Title/Abstract]) OR (Isometric Exercises[Title/Abstract]) OR (Exercise, Aerobic [Title/Abstract]) OR (Aerobic Exercise [Title/Abstract]) OR (Exercises, Aerobic [Title/Abstract]) OR (Exercise Trainings [Title/Abstract]) OR (Training, Exercise [Title/Abstract]) OR (Trainings, Exercise [Title/Abstract]) AND (Randomized Controlled Trial [Title/Abstract]) OR (Randomized [Title/Abstract]) OR (Randomized Controlled Trials [Title/Abstract]) OR (Randomised Controlled Trials [Title/Abstract]) OR (RCT [Title/Abstract]) OR (RCTs [Title/Abstract])

Cochrane library search strategy:

#1 MeSH descriptor: [Students] explode all trees
#2 (Student):ti,ab,kw (Word variations have been searched)
#3 (School Enrollments):ti,ab,kw (Word variations have been searched)
#4 (Enrollment, School):ti,ab,kw (Word variations have been searched)
#5 MeSH descriptor: [Child] explode all trees
#6 (Children):ti,ab,kw (Word variations have been searched)
#7 MeSH descriptor: [Adolescent] explode all trees
#8 (Adolescents):ti,ab,kw (Word variations have been searched)
#9 (Adolescence):ti,ab,kw (Word variations have been searched)
#10 (Teens):ti,ab,kw (Word variations have been searched)
#11 (Teenagers):ti,ab,kw (Word variations have been searched)
#12 (Youths):ti,ab,kw (Word variations have been searched)
#13 (Adolescents, Female):ti,ab,kw (Word variations have been searched)
#14 (Female Adolescent):ti,ab,kw (Word variations have been searched)
#15 (Male Adolescents):ti,ab,kw (Word variations have been searched)
#16 #1 or #2 or #3 or #4 or #5 or #6 or #7 or #8 or #9 or #10 or #11 or #12 or #13 or #14 or #15
#17 MeSH descriptor: [Mental health] explode all trees
#18 (Health, Mental):ti,ab,kw (Word variations have been searched)
#19 (Mental Hygiene):ti,ab,kw (Word variations have been searched)
#20 (Nursing, Mental Health):ti,ab,kw (Word variations have been searched)
#21 (Nursing, Psychiatric):ti,ab,kw (Word variations have been searched)
#22 (Nursing, Psychosocial):ti,ab,kw (Word variations have been searched)
#23 (Rehabilitation, Psychiatric):ti,ab,kw (Word variations have been searched)
#24 (Mental Health Rehabilitation):ti,ab,kw (Word variations have been search
#25 (Rehabilitation, Mental Health):ti,ab,kw (Word variations have been searched)
#26 (Psychosocial Rehabilitation):ti,ab,kw (Word variations have been searched)
#27 #17 or #18 or #19 or #20 or #21 or #22 or #23 or #24 or #25 or #26
#28 MeSH descriptor: [Pediatric obesity] explode all trees
#29 (Obesity, Pediatric):ti,ab,kw (Word variations have been searched)
#30 (Childhood Onset Obesity):ti,ab,kw (Word variations have been searched)
#31 (Obesity, Childhood Onset):ti,ab,kw (Word variations have been searched)
#32 (Obesity in Childhood):ti,ab,kw (Word variations have been searched)
#33 (Child Obesity):ti,ab,kw (Word variations have been searched)
#34 (Obesity, Child):ti,ab,kw (Word variations have been searched)
#35 (Childhood Obesity):ti,ab,kw (Word variations have been searched)
#36 (Infant Overweight):ti,ab,kw (Word variations have been searched)
#37 (Infantile Obesity):ti,ab,kw (Word variations have been searched)
#38 (Infant Obesity):ti,ab,kw (Word variations have been searched)
#39 (Childhood Overweight):ti,ab,kw (Word variations have been searched)
#40 (Adolescent Overweight):ti,ab,kw (Word variations have been searched)
#41 (Adolescent Obesity):ti,ab,kw (Word variations have been searched)
#42 (Obesity, Adolescent):ti,ab,kw (Word variations have been searched)

#43 #28 or #29 or #30 or #31 or #32 or #33 or #34 or #35 or #36 or #37 or #38 or #39 or #40 or #41 or #42
#44 MeSH descriptor: [Exercises] explode all trees
#45 (Physical Activity):ti,ab,kw (Word variations have been searched)
#46 (Physical Activities):ti,ab,kw (Word variations have been searched)
#47 (Exercise, Physical):ti,ab,kw (Word variations have been searched)
#48 (Acute Exercise):ti,ab,kw (Word variations have been searched)
#49 (Exercise, Acute):ti,ab,kw (Word variations have been searched)
#50 (Exercise, Isometric):ti,ab,kw (Word variations have been searched)
#51 (Isometric Exercises):ti,ab,kw (Word variations have been searched)
#52 (Exercise, Aerobic):ti,ab,kw (Word variations have been searched)
#53 (Aerobic Exercise):ti,ab,kw (Word variations have been searched)
#54 (Exercise Training):ti,ab,kw (Word variations have been searched)
#55 (Training, Exercise):ti,ab,kw (Word variations have been searched)
#56 #44 or #45 or #46 or #47 or #48 or #49 or #50 or #51 or #52 or #53 or #54 or #55
#57 #16 AND #27 AND #56
#58 #16 AND #43 AND #56

Embase search strategy:

#1 "Child"/exp OR "child"
#2 "Adolescents"/exp OR "students"
#3 "Teenagers"/exp OR "students"
#4 "Mental health"
#5 "Attention deficit hyperactivity disorder"
#6 "Anxiety"
#7 "Depression"
#8 "Autism Spectrum Disorder"
#9 "Obesity"
#10 "Physical Activity"
#11 "Exercise"
#12 "Aerobic Exercise"
#13 "Exercise Training"
#14 #1 OR #2 OR #3
#15 #4 OR #5 OR #6 OR #7 OR #8
#16 #10 OR #11 OR #12 OR #13
#17 #14 AND #15 AND #16 ([meta analysis]/lim OR [randomized controlled trial]/lim)
#18 #14 AND #9 AND #16 ([meta analysis]/lim OR [randomized controlled trial]/lim)
