# Supplementary material for: Meta-analysis and systematic review of physical activity on neurodevelopment disorders, depression, and obesity among children and adolescents
Source: Front Psychol. 2022 Nov 30;13:940977. doi: 10.3389/fpsyg.2022.940977 (PMC9747947; doi:10.3389/fpsyg.2022.940977)
Supplement: Supplementary Table 1 — Demographic characteristics of the included studies and their participants. [file Table_1.DOCX]

| **Table.1 Demographic characteristics of the included studies and their participants** | | | | |
| --- | --- | --- | --- | --- |
| Characteristics of the 31 included studies | No(%)of studies |  | Characteristics of participants of the 31 included studies | No(%)of studies |
| **Publication year** | **31(100%)** |  | **Baseline age** | **31(100%)** |
| 2000-2005 | 1(3.23%) |  | Children | 17(54.84%) |
| 2006-2010 | 9(29.03%) |  | Adolescents | 13(41.94%) |
| 2011-2015 | 17(54.84%) |  | Both | 1(3.23%) |
| 2016- | 4(12.90%) |  | **Proportion boys(%)** | **31(100%)** |
| **Study intervention duration (weeks)** | **31(100%)** |  | ≥50 | 24(77.42%) |
| 1-8 | 9(29.03%) |  | ＜50 | 4(12.90%) |
| 9-12 | 9(29.03%) |  | NR | 3(9.68%) |
| 13-16 | 4(12.90%) |  | **Education** | **31(100%)** |
| 17- | 7(22.58%) |  | Primary | 17(54.84%) |
| NR/Unclear | 2(6.45%) |  | Middle | 7(22.58%) |
| **Geographical region** | **31(100%)** |  | High | 6(19.35%) |
| America | 9(29.03%) |  | Unclear | 1(3.23%) |
| Europe | 6(19.35%) |  |  |  |
| Asia | 14(45.16%) |  |  |  |
| Other | 2(6.45%) |  |  |  |
| **Diagnosis criteria** | **31(100%)** |  |  |  |
| **Mental disorders** | **13(41.94%)** |  |  |  |
| DSM-IV/DSM-IV-TR | 8(25.81%) |  |  |  |
| DSM-V | 1(3.23%) |  |  |  |
| Multi-method | 2(6.45%) |  |  |  |
| Other | 2(6.45%) |  |  |  |
| **Obese** | **18(58.06%)** |  |  |  |
| Percentile BMI | 16(51.61%) |  |  |  |
| NR | 1(3.23%) |  |  |  |
| Multi-method | 1(3.23%) |  |  |  |
| **Disease type** | **31(100%)** |  |  |  |
| **Mental disorders** | **13(41.94%)** |  |  |  |
| Depression | 4(12.90%) |  |  |  |
| ASD | 5(16.13%) |  |  |  |
| ADHD | 4(12.90%) |  |  |  |
| **Obese** | **18(58.06%)** |  |  |  |
| Obesity | 12(38.71%) |  |  |  |
| Overweight | 6(19.35%) |  |  |  |
| **Quality of study(ROB)** | **31(100%)** |  |  |  |
| Low | 27(87.10%) |  |  |  |
| High | 4(12.90%) |  |  |  |
| **Sample size(n)** | **31(100%)** |  |  |  |
| 1-20 | 4(12.90%) |  |  |  |
| 21-30 | 10(32.26%) |  |  |  |
| 31-40 | 9(29.03%) |  |  |  |
| 41- | 8(25.81%) |  |  |  |

ASD, Autism Spectrum Disorder; ADHD, Attention deficit hyperactivity disorder; BMI, Body mass index; DSM-IV/DSM-IV-TR, Diagnostic and Statistical Manual of Mental Disorders-Fourth Edition/Text Revision; DSM-V, Diagnostic and Statistical Manual of Mental Disorders-Fifth-Edition; NR, Not reported; ROB, Risk of bias.
