# Supplementary material for: Meta-analysis and systematic review of physical activity on neurodevelopment disorders, depression, and obesity among children and adolescents
Source: Front Psychol. 2022 Nov 30;13:940977. doi: 10.3389/fpsyg.2022.940977 (PMC9747947; doi:10.3389/fpsyg.2022.940977)
Supplement: Supplementary Table 2 — The demographic characteristics of included studies. [file Table_2.docx]

**Table.2 The demographic characteristics of included studies**

| Source | Diagnose | Sample size | | Proportion of girls(%) | Age Mean ± sd/range | | Grade | Intervention duration (Weeks) | Potential confoundings | Area | Outcomes measurements |
| --- | --- | --- | --- | --- | --- | --- | --- | --- | --- | --- | --- |
|  |  |  |  |  |  |  |  |  |  |  |  |
|  |  | PA | CG |  | PA | CG |  |  |  |  |  |
| Borgi, 2015 | ASD | 15 | 13 | All boys | 8.60 ± 1.70 | | Primary | 24 | * | Italy | VABD, TOL |
| Bahrami, 2015 | ASD | 15 | 15 | 13.33 | 9.20 ± 3.32 | 9.06 ± 3.33 | Primary | 14 | NR | USA | CSGARS-2 |
| Bass, 2009 | ASD | 19 | 15 | 14.71 | 6.95 ± 1.67 | 7.73 ± 1.65 | Primary | 12 | NR | USA | SRS |
| Movahedi, 2013 | ASD | 15 | 15 | 13.33 | 9.54 ± 3.43 | 9.06 ± 3.33 | Primary | 14 | NR | Iran | CSGARS-2 |
| Kadri, 2019 | ADHD | 20 | 20 | 10 | 14.50 ± 3.50 | 14.20 ± 3.00 | Middle | 78 | * | Tunisia(Africa) | CBT, CWIT |
| Pan, 2014 | ADHD | 12 | 12 | All boys | 9.63 ± 2.48 | 9.38 ± 2.69 | Primary | 12 | Fifteen were on medication and 5 manifested diagnosed associated conditions | Taiwan(China) | BOT-2 |
| Memarmoghaddam, 2016 | ADHD | 19 | 17 | All boys | 8.31 ± 1.29 | 8.29 ± 1.31 | Primary | 8 | * | Iran | CBCL, CPMST, Go-No-Go test |
| Chang, 2012 | ADHD | 20 | 20 | 7.5 | 10.45 ± 0.95 | 10.42 ± 0.87 | Primary | NR | * | China | Stroop test, WCST |
| Choi, 2014 | ADHD | 13 | 17 | All boys | 15.80 ± 1.70 | 16.00 ± 1.20 | High | 6 | * | Korea | K-ARS |
| Branch, 2011 | Depression | 20 | 20 | NR | NR | | High | 8 | Some of the participants were on medication | Iran | CDI-2 |
| Hughes, 2013 | Depression | 16 | 14 | 50 | 16.30-17.90 | 16.30-17.60 | primary& middle& high | 52 | * | USA | C-GAS, F-GAS, QIDS |
| Roshan, 2011 | Depression | 12 | 12 | All girls | 16.91 ± 1.03 | 16.83 ± 0.82 | High | 6 | * | Iran | Ham-D |
| Carter, 2015 | Depression | 44 | 43 | 78.16 | 15.40 ± 1.00 | 15.40 ± 0.90 | High | 26 | * | UK | CDI |
| Alberga, 2013 | Obese | 12 | 7 | 31.58 | 10.00 ± 2.00 | 10.00 ± 1.00 | Primary | 12 | * | Canada | BMI, FMI, LBMI |
| Ounis, 2010 | Obese | 16 | 16 | 53.13 | 13.40 ± 0.40 | 13.20 ± 0.60 | Middle | 8 | * | Fance | BMI, Body fat, WC, SBP, DBP, RHR, HDL. |
| Chen, 2016 | Obese | 25 | 25 | 44 | 12.64 ± 0.70 | 12.84 ± 0.75 | High | 12 | * | Taiwan(China) | BMI, Body fat |
| Davis, 2012 | Obese | 77 | 78 | 57.05 | 9.30 ± 0.90 | 9.40 ± 1.10 | Primary | 10-15 | * | USA | BMI, Body fat, VO_2_ |
| Lambert, 2009 | Obese | 22 | 22 | 36.36 | 9.10 ± 1.40 | 8.80 ± 1.60 | Primary | 12 | * | Switzerland | BMI, Body fat, HDL, LDL |
| Mer, 2009 | Obese | 16 | 15 | 54.84 | 13.70 ± 2.00 | 13.60 ± 2.20 | Middle | 13 | NR | Sweden | BMI, VO_2_ |
| Wittmeier, 2015 | Obese | 32 | 36 | 70.59 | 15.10 ± 1.80 | 15.20 ± 1.70 | Middle | 24 | * | Canada | BMI, Body fat, SBP, DBP, VO_2_ |
| Karacabey, 2009 | Obese | 20 | 20 | NR | 11.20 ± 0.80 | 11.80 ± 0.50 | Primary | 12 | * | Turkey | BMI, HDL, LDL |
| Kelly, 2004 | Obese | 10 | 10 | 55 | 11.00 ± 0.63 | 11.00 ± 0.71 | Primary | 8 | NR | USA | BMI, SBP, DBP, HDL, LDL |
| Eun Sung Kim, 2006 | Obese | 14 | 12 | All boys | 17.00 ± 0.11 | | High | 6 | * | Korea | BMI, SBP, DBP, HDL, LDL |
| Hyun Jun Kim, 2008 | Obese | 8 | 9 | All boys | 11 years old | | Primary | 12 | * | Korea | BMI, PBF |
| Lau, 2014 | Obese | 21 | 12 | NR | 9.90 ± 0.90 | 10.60 ± 0.60 | Primary | 6 | * | Korea | BMI, HIIE, LIIE, MAS, YYIET |
| Meyer, 2006 | Obese | 33 | 34 | 49.25 | 13.70 ± 2.10 | 14.10 ± 2.40 | Primary | 24 | NR | Germany | BMI, Body fat, HDL, LDL |
| Shaibi, 2006 | Obese | 11 | 11 | NR | 15.10 ± 0.50 | 15.60 ± 0.60 | Middle | 16 | NR | USA | BMI, Body fat, VO_2_ |
| Seo, 2012 | Obese | 10 | 10 | All boys | 14.70 ± 0.48 | 14.60 ± 0.96 | Middle | 8 | Participants who had hypertension were using medication | Korea | BMI, Body fat, BMR |
| Nobre, 2016 | Obese | 40 | 19 | All boys | 9.80 ± 0.90 | 9.90 ± 1.10 | Primary | 12 | NR | Brazil | BMI |
| Park, 2012 | Obese | 15 | 14 | 51.72 | 12.10 ± 0.10 | 12.20 ± 0.10 | Primary | 12 | * | Korea | BMI, WC, SBP, DBP |
| Schranz, 2013 | Obese | 30 | 26 | All boys | 14.90 ± 1.40 | 15.10 ± 1.60 | Middle | 24 | NR | Australia | BMI, PBF |

ASD, Autism Spectrum Disorder; ADHD, Attention deficit hyperactivity disorder; BMI, Body mass index; BMR, basal metabolic rate; BOT-2, Bruininks-Oseretsky test of motor proficiency, second edition; CBCL, Child behavior checklist; CBT, Color block test; CDI-2, Children’s depression inventory-2; CG, Control group; C-GAS, Children’s Global Assessment Scale; CPMST, Cognitive performance measures stroop test; CSGARS-2, communication subscale of Gilliam Autism Rating Scale-Second Edition; CWIT, Color-word interference test; DBP, Diastolic blood pressure; F-GAS, Family Global Assessment Scale; FMI, fat mass index; H, High; Ham-D, Hamilton rating scale for depression; HDL, High-density lipoprotein; HIIE, Higher intensity intermittent exercise group; K-ARS, Dupaul attention deficit hyperactivity disorder rating scale–Korean version; L, Low; LBMI, lean body; LDL, Low Density Lipoprotein; LIIE, lower intensity intermittent exercise group; MAS, maximal aerobic speed; PA, Physical exercise; PBF, Percentage body fat; QIDS, Quick Inventory for Depressive Symptomatology; RHR, Resting heart rate; ROB, Risk of bias; SBP, Systolic blood pressure; SRS, Social responsiveness scale; TOL, Tower of London; VABS, Vineland Adaptive Behavior Scale; VO2, oxygen consumption; WC, Waist circumference; WCST, Wisconsin card sorting test; YYIET, Yo-Yo Intermittent Endurance Test.

*, Participants receiving medications, with a history of recent intercurrent illness, or who were currently participating in any organized physical

activity training programs were excluded from the study.
